# School alienation and students’ perceptions of teacher justice

**DOI:** 10.1007/s11218-026-10200-6

**Published:** 2026-03-20

**Authors:** Angela Aegerter, Tina Hascher, Julia Mori

**Affiliations:** Department of Research in School and Instruction, Institute of Educational Science, University of Bern, Fabrikstrasse 8, 3012 Bern, Switzerland

**Keywords:** School alienation, Teacher justice, Supportive school environment, Qualitative analysis

## Abstract

School alienation, understood as a negative attitude toward school-related activities and the school community, increases across schooling and poses challenges for teachers and educational professionals. In this process, students’ perceptions of teacher justice play an important role. Drawing on data from 15 focus groups with *N* = 134 Swiss students (four schools from two school districts) in the binational project School Alienation in Switzerland and Luxembourg (SASAL), this study investigates how aspects of school alienation are associated with perceived teacher injustice. At two measurement points covering the transition from primary to lower secondary education, students were asked to describe situations of school alienation. Structured content analysis revealed several domains in which experiences central to school alienation co-occurred with perceived injustice, including the student–teacher relationship, handling of peer conflicts, learning processes, assessments, disciplinary measures, allocation, and transition. Distinct patterns emerged between school levels, reflecting performance heterogeneity in primary classes and the subject-teacher system in lower secondary education as relevant to the association between school alienation and teacher injustice. The findings emphasize the value of fostering a just, safe, and supportive school environment and building trusting relationships to positively shape students’ perceptions of school experiences and mitigate school alienation.

## Introduction

The school environment is an important social sphere for learning and experience and is considered pivotal in the socialization of children and adolescents (Donat et al., 2014; Peter et al., 2013a). It is thus concerning that negative attitudes toward school increase throughout the academic journey, become more pronounced at the lower secondary level, and can cause school alienation (Hascher & Hagenauer, 2010). School alienation, defined as a set of negative attitudes toward the social and academic domains of school that lead to a process of alienation from school-related activities such as learning and the school community, including teachers and peers, has multifaceted consequences (Hascher & Hadjar, 2018, 2021). School alienation is likely to negatively affect students’ academic success and, in turn, undermine the fundamental goals of education (Çağlar, 2013). Hence, factors that contribute to alienation need to be identified to enable educational institutions to proactively address school alienation and its adverse effects (Berti et al., 2016; Çağlar, 2013). School alienation research suggests that schools that provide a just, supportive, safe, and enjoyable learning environment support student well-being and prevent school alienation (Çağlar, 2013; Grecu et al., 2022; Hascher & Hadjar, 2018, 2021; Hascher & Hagenauer, 2020). A positive learning environment and students’ perceptions of teacher justice can be construed as beneficial psychosocial resources (Berti et al., 2016; Peter and Dalbert 2013) that mitigate feelings of alienation (Çağlar, 2013; Looker, 2022). However, little is known about the specific role that teacher injustice plays in student alienation and how students explain their experiences of teacher injustice in the context of school alienation in primary and lower secondary classrooms. Our study thus aims to (1) gain a deeper understanding of the relation between school alienation and teacher justice to enhance the understanding of supportive student–teacher relationships and teacher behavior, (2) offer insights that can inform practices aimed at fostering a just environment in schools, and (3) ultimately contribute to the prevention of school alienation. The topic of school alienation is important for teachers and other professionals in education due to its potential impact on learning difficulties, behavioral problems, and educational trajectories. Given that feelings of alienation from school can arise in diverse cultural and educational contexts, the topic holds international relevance and demands cross-national attention (Brown et al., 2003b; Ditton 2010; Hascher & Hadjar 2018, 2021; Hascher & Hagenauer 2010; Morinaj et al., 2023). Awareness and recognition of school alienation processes can inform effective multiprofessional collaboration between schools and families and thus promote the creation of a supportive learning environment that addresses students’ needs.

### School alienation

The concept of alienation can be traced back to Karl Marx (1844). From a philosophical perspective, Marx described alienation from human productive activities within the context of labor. Alienation was described as a disturbed relationship between individuals and the products of their labor and ultimately with the productive activity. This disruption has significant effects on the individual that become evident in feelings of detachment from and personal insignificance regarding the results of labor (Hascher & Hadjar, 2018, 2021; Sidorkin, 2004).

The negative evaluation of the activity and the feelings of detachment also informed the construct of school alienation. Hascher and Hadjar (2018) conducted an extensive literature review of prevailing concepts of alienation and related empirical studies. Their review led them to formulate a definition of school alienation that acknowledges its process-oriented nature, delimits distinct domains, and clearly distinguishes school alienation from its consequences (Hascher & Hadjar, 2018, 2021). School alienation stems from a general negative attitude toward school that evolves through a succession of adverse experiences throughout a student’s school career. Hence, students develop negative perceptions of school as a learning and living environment within the cognitive component of school alienation, and experience negative emotions, accompanied by a diminished sense of belonging, within its affective component. School alienation, understood as a negative attitude, is thus clearly distinguished from individual student behavior, which is an expression or consequence of school alienation (Hascher & Hadjar, 2018, 2021). Furthermore, school alienation seems context-dependent and should be interpreted within the specific educational setting, as schools provide a diverse array of individual experiences (Hascher & Hadjar, 2018, 2021). Factors such as institutional structures, school culture, and especially the manner in which schools address various challenges can impact the alienation process significantly (Brown et al., 2003a, 2003b; Stamm & Holzinger-Neulinger, 2012).

**Fig. 1:**
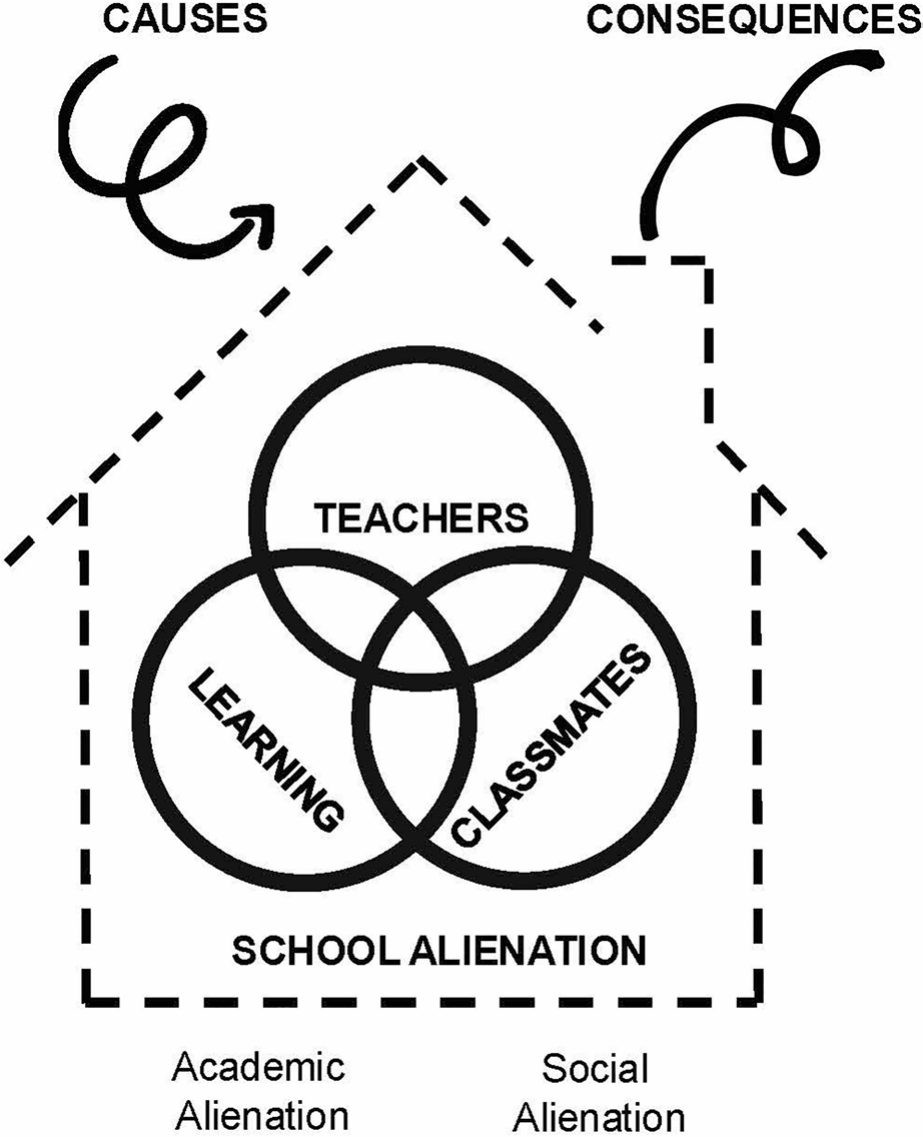
Conceptual model of school alienation (Hascher & Hadjar, 2018), adapted by the authors

The framework of Hascher and Hadjar (2018) consequently distinguishes between academic and social alienation, enabling a categorization into three domains: “(1) learning as the main objective of schooling; (2) teachers as educators and mentors who both represent the school authority (professional level) and form a part of the social environment; and (3) classmates as a social peer community (Hascher & Hadjar, 2018, p. 179)”. The multidimensional nature of the construct captures the diverse forms in which school alienation may emerge. Alienation may occur within specific domains of school life—learning, teachers, or classmates—or extend across multiple domains, leading to complex interactions (Hascher & Hadjar, 2018, 2021). Figure 1 provides a visual synthesis of this multidimensional structure, which also illustrates how the domains may overlap in shaping school alienation.

The concept of school alienation encompasses students’ negative attitudes that impact their educational achievement and success (Brown et al., 2003a; Hascher & Hadjar, 2018, 2021). Research conducted at the primary school level indicates a progressive increase in critical and negative attitudes toward school across the primary school years, accompanied by declines in enjoyment of learning activities and educational self-concept (Hascher et al., 2011). Such tendencies persist and intensify upon the transition to lower secondary education, culminating in various forms of school alienation, including low engagement in school-related activities, disruption during lessons, deviant behavior, truancy, absenteeism, and an increased risk of dropout (Archambault et al., 2009; Brown et al., 2003a; Hascher & Hagenauer, 2010; Morinaj et al., 2020). During the transition to lower secondary education, students encounter a myriad of stressors associated with feelings of excitement, nervousness, and perceived threats (Stern, 2012; Symonds & Hargreaves, 2016). Teachers play a key role in providing individualized and attentive support (Grecu et al., 2019; Marcin et al., 2020). However, research indicates a decline in student attachment to teachers during this period, which is associated with more negative perceptions of teachers and therefore the loss of a significant source of emotional support (Eccles et al., 1996; Symonds & Hargreaves, 2016). At the same time, the importance of peer relationships increases and exerts substantial influence on the process of school alienation (Morinaj et al., 2023). The post-transition phase is frequently marked by more negative perceptions of school and heightened concerns about social status among peers (Eccles et al., 1996). Social integration and a sense of belonging to the school community significantly affect students’ general life satisfaction (Aegerter et al., 2023). Therefore, fostering positive relationships with both peers and teachers emerges as imperative in educational contexts, particularly as the school social climate is strongly associated with school alienation (Buzzai et al., 2022; Looker, 2022; Schmid et al., 2021). Considering the processual nature of school alienation and its substantial role as a stressor in the lives of young people, there is a collective responsibility on the part of teachers, school psychologists, and educational networks to address this challenging educational issue (Brown et al., 2003a, 2003b; Morinaj & Hascher, 2019).

As teachers are involved in all three core domains of school alienation—learning processes, teacher–student relationships, and the peer environment—their behavior is a central potential source of alienation. Prior studies indicate that perceptions of teacher (in-)justice are closely associated with students’ attitudes toward school (Çağlar, 2013; Hascher & Hadjar, 2018, 2021; Marcin et al., 2020). Teacher justice therefore provides an important conceptual perspective for understanding how experiences in these domains may shape the development of school alienation.

### Teacher justice

Justice as a value has a universal character that holds significance for individuals across all life stages and may be regarded as a basic human need (Donat et al., 2012; Taylor, 2003, 2017), thereby underscoring the international importance of the topic across diverse cultural and regional contexts. Nevertheless, conceptions of justice are inherently context-dependent, as individuals’ subjective perceptions are shaped by the social environments in which they are situated (Looker, 2022; Peter et al., 2013b).

Although often used interchangeably, *justice* and *fairness* represent distinct constructs. Recent work has clarified that justice is descriptive—referring to the extent to which justice principles or norms are upheld—whereas fairness is evaluative and reflects individuals’ global judgments of appropriateness, without differentiating among justice dimensions (Colquitt et al., 2005; Colquitt & Rodell, 2015; Cropanzano et al., 2015; Zhaleh et al., 2025). Justice perceptions therefore inform and predict perceived fairness (Cropanzano et al., 2015; Zhaleh et al., 2025).

Organizational justice frameworks have been widely applied in education and have proven useful for explaining students’ experiences (for an overview, see Rasooli et al., 2018). In school settings, justice perceptions are especially salient in areas such as grading, instructional support, autonomy, differentiation and disadvantage, discipline and classroom management, and teacher–student interactions (Bakchich et al., 2025; Chory, 2007; Čopková, 2020; Dalbert et al., 2007; Geenen et al., 2025; Grazia, 2025; Grazia et al., 2021; Helm et al., 2020; Pnevmatikos & Trikkaliotis, 2012; Pretsch et al., 2016; Sabbagh, 2021; Thorkildsen et al., 1994; Xu & Chen, 2023). Overall, justice in school contexts can be categorized into the domains of learning, teaching, assessment, and interaction (Rasooli et al., 2018).

Within educational settings, teachers are pivotal figures who influence students’ perceptions of justice through their conduct and actions in the classroom (Resh & Sabbagh, 2014). Dalbert and colleagues conceptualize teacher justice as students’ subjective evaluations of teachers’ actions (Peter & Dalbert, 2010). Such judgments reflect intuitive, individually constructed interpretations rather than objective features of a situation (Dalbert, 2004; Mikula, 2005; Peter & Dalbert, 2010). Thus, teacher justice refers to students’ subjective, individual perceptions of justice in teacher–student interactions (Dalbert & Stoeber, 2006).

When students perceive their teachers as just, two key psychological processes are initiated: Firstly, students perceive themselves as members of a just community, which fosters confidence in the expectation of just treatment in the future. Second, it motivates the commitment to act justly to ensure that the just community is maintained (Dalbert, 2013). These processes align with *group value theory* (Lind & Tyler, 1988; Tyler & Lind, 1990), a psychological account of justice commonly applied in discussions of procedural justice. The theory posits that justice judgments are shaped less by self-interest than by perceptions of one’s status and group membership (Lind & Tyler, 1988; Tyler et al., 1996; Tyler & Lind, 1990). Procedures are viewed as just when they affirm core group values—such as recognition, belonging, participation, solidarity, and stable authority relations—and such perceptions influence individuals’ evaluations of the group and its authorities. Just treatment fosters commitment and loyalty, whereas injustice evokes negative emotions and, over time, shapes self-concept and long-term group relations (Lind & Tyler, 1988; Tyler et al., 1996; Umlauft et al., 2013).

In school settings, these mechanisms are equally operative. Justice experiences enhance students’ sense of belonging, a central determinant of motivation, well-being, and behavior (Donat et al., 2013; Tyler & Lind, 1990; Umlauft et al., 2013). Belonging, in turn, promotes adherence to norms and constructive participation in classroom life (Blader & Tyler, 2009; Donat et al., 2013). Conversely, experiences of injustice may contribute to deviant behavior, rule violations, socially disruptive actions, bullying, deceit, or delinquent behaviors (Donat et al., 2013). Because school is a significant social arena, justice-related experiences have lasting implications for students’ personality development, institutional trust, social integration, and feelings of societal exclusion (Umlauft et al., 2013). Given the far-reaching effects of perceived injustice for students’ motivation, attitudes, emotions, educational opportunities, and behavior (Helm et al., 2020; Resh & Sabbagh, 2009), perceptions of teacher justice are essential for social, emotional, and academic development (Dalbert, 2013; Donat et al., 2014; Sabbagh, 2021).

Experiences of injustice may arise in various school situations, particularly those involving behavior regulation, interpersonal interactions, and performance assessment (Peter et al., 2013b). Researchers have shown a strong interest in investigating which specific actions of teachers are subjectively perceived as just or unjust by students (Peter et al., 2013b).

Conceptually, teacher justice builds on *organizational justice theory* (Adams, 1965; Bies & Moag, 1986; Leventhal, 1976, 1980), which examines how individuals perceive justice within organizational settings (Cropanzano & Greenberg, 1997). Organizational justice encompasses evaluations of justice inherent in outcome distributions (distributive justice), of the procedures through which these outcomes are determined (procedural justice), and of the interpersonal treatment and informational adequacy provided throughout decision-making processes (interpersonal and informational justice) (Greenberg & Colquitt, 2005; Lind & Tyler, 1988). Transferred to school settings, these three dimensions can be described as follows:

First, *interactional justice* concerns the appropriateness of teachers’ interpersonal and communicative conduct (Dalbert 2013; Lotz et al., 2013; Peter et al., 2013b). It comprises interpersonal justice, referring to respectful, polite, and dignified treatment, and informational justice, which involves transparent, accurate, and timely communication about decisions and procedures (Dalbert, 2013; Lotz et al., 2013). With respect to interpersonal justice, it is the teachers’ responsibility to initiate and exemplify appropriate behavior in social interactions. Classroom interactions have been shown to be improved by establishing explicit agreements on acceptable conduct and documenting them in written form (Dalbert, 2013). Regarding informational justice, openness and transparency are essential; both factually relevant information (e.g., decision criteria) and personally meaningful information (e.g., students’ emotional states or perspectives) must be communicated (Peter et al., 2013b). Interactional justice is particularly critical in assessment situations, where respectful communication fosters students’ trust, commitment, and intrinsic motivation (Chory-Assad & Paulsel, 2004; Kazemi, 2016).

Second, *procedural justice* pertains to the appropriateness of a procedure as the foundation for decision-making by teachers and the associated perception of control by students (Lotz et al., 2013; Peter et al., 2013b). For example, this applies to decisions about assessments, rewards, and disciplinary measures (Peter et al., 2013b). Students perceive procedures as just when they are consistent, unbiased, accurate, corrigible, representative, and ethical—criteria aligned with Leventhal’s framework (Dalbert, 2004, 2013; Leventhal, 1980). Justice perceptions also depend on whether students’ interests are considered throughout the process, whether they are given opportunities to voice concerns or objections, and whether decisions are made at a professional standard (Dalbert, 2013). The more criteria a procedure fulfills, the higher its perceived justice (Leventhal, 1980); violations, by contrast, elicit perceptions of injustice (Peter et al., 2013b). However, the relative weight of specific criteria across different school situations remains insufficiently understood and requires further investigation (Peter et al., 2013b). Students’ evaluations of instructional practices appear pluralistic and context-dependent, underscoring the need for dialogue about justice in the classroom (Thorkildsen et al., 1994). Further empirical research indicates that transparency and the consistent use of criterion-referenced assessment are central to strengthening perceptions of justice in performance evaluation. Given that the teaching principle of differentiation is also considered a legitimate principle of justice, any unequal treatment must be made transparent to students to counteract perceptions of injustice (Dalbert, 2013; Pnevmatikos & Trikkaliotis, 2012).

Third, *distributive justice* refers generally to whether conditions and goods are distributed in a just manner—such as grades or educational opportunities (Chory-Assad & Paulsel, 2004; Dalbert, 2013; Deutsch, 1975). Deutsch (1975) distinguishes three central principles of distributive justice: equity, equality, and need. The equity principle holds that outcomes should be proportional to performance; for example, that evaluations should reflect students’ individual effort (Dalbert, 2004; Deutsch, 1975). The equality principle stresses the preservation of harmonious social relationships and mandates equal treatment of all parties involved (Deutsch, 1975), such as awarding identical grades for equivalent performance (Dalbert, 2004). The need principle underscores individual developmental requirements and justifies need-oriented, unequal distributions (Deutsch, 1975), for instance, in favor of students whose grade progression may be at risk (Dalbert, 2004). Which principle is regarded as just depends on situational conditions and is shaped by social group norms (Dalbert, 2004; Deutsch, 1975). Empirical findings indicate that, in grading contexts, students generally favor equality over equity or need (Dalbert, 2004).

### Relation between school alienation and teacher justice

School alienation emerges when students repeatedly experience school as unresponsive, unsupportive, or emotionally distant. These processes unfold across the domains of learning, teachers, and classmates, in which negative experiences accumulate over time and contribute to cognitive and emotional distancing from school (Hascher & Hadjar, 2018, 2021; Hascher & Hagenauer, 2010). Teachers play a central role in these processes because they shape students’ daily academic and interpersonal experiences more directly than any other actor in the school context (Marcin et al., 2020; Morinaj & Hascher, 2019). Their behavior influences whether students experience school as meaningful, respectful, and just—or, conversely, as arbitrary and unjust. Research shows that supportive and stable teacher–student interactions foster meaningful learning experiences and strengthen students’ sense of belonging, whereas a lack of supportive or respectful teacher behavior is associated with exclusion, social problems, and increased school alienation (Marcin et al., 2020).

Psychological theories further illuminate this connection. According to group-value theory (Lind & Tyler, 1988; Tyler & Lind, 1990), justice experiences serve as social signals that communicate recognition, belonging, and respect. Unjust treatment instead conveys devaluation and exclusion. These social appraisals align closely with the core antecedents of school alienation, which is characterized by diminished belonging, trust, and perceived relevance (Hascher & Hadjar, 2018, 2021). Empirical studies confirm this link: perceived teacher injustice predicts reduced school belonging and feelings of exclusion (Donat et al., 2018; Umlauft & Dalbert, 2017), and unjust instructional or relational practices are associated with components of alienation such as meaninglessness and powerlessness (Çağlar, 2013). Moreover, Marcin et al. (2020) demonstrate that perceptions of teacher injustice undermine students’ psychological needs and contribute specifically to alienation from learning, underscoring the role of justice as a proximal driver of alienation processes.

Taken together, we argue that the two constructs are related and mutually influential, with a positive school environment helping to reduce both school alienation and perceptions of teacher injustice (Berti et al., 2016; Çağlar, 2013; Hascher & Hagenauer, 2010; Hascher et al., 2018). Therefore, we aim to investigate which aspects of school alienation are related to perceived teacher justice.

### The present study

This qualitative study aimed to explore in depth the aspects of school alienation that relate to students’ perceptions of teacher (in)justice. We assumed that students’ perceptions of teacher injustice are important elements in the process of school alienation. Accordingly, one research objective was to identify the specific aspects that characterize this association. Given the mutual influence of the two constructs, we proposed that students’ perceptions of teacher justice are both a precursor and an outcome of school alienation. Furthermore, we expected students’ perceptions to differ between primary and lower secondary school students due to notable changes in the school environment and schooling following this transition in the Swiss educational system: In Switzerland, the transition to lower secondary level marks a significant turning point in a student’s academic trajectory. Unlike in primary school, students in lower secondary school are taught within a highly stratified school system in which they are allocated to different academic tracks with varying requirements based on their academic achievement at the end of primary school. Due to Switzerland’s federal structure, school organization varies across municipalities, so that the transition often entails new class compositions and the implementation of a subject-teacher system (Behrens, 2016; Hascher & Morinaj, 2023). Thus, the research questions (RQ) guiding this study are:

1. How are the three domains of school alienation generally associated with students’ perceptions of teacher (in)justice? (RQ1)
2. Which dimensions of teacher (in)justice are related to the three domains of school alienation? (RQ2)
3. How do these associations differ between primary and lower secondary school students? (RQ3)

By examining the association between school alienation and teacher justice, this study offers practical implications for raising awareness among teachers, school psychologists, and other educational professionals. The identified aspects provide valuable guidance for addressing and, more importantly, preventing school alienation by drawing greater attention to justice-related factors.

## Method

### Setting and sample

The qualitative data used were part of a binational mixed-methods research project titled School Alienation in Switzerland and Luxembourg (SASAL; 2015–2019). This longitudinal research project was dedicated to investigating the nature, precursors, and consequences of school alienation by focusing on the subjective attitudes and experiences of students regarding school and learning. Within the SASAL research design, an annual questionnaire survey was administered to two cohorts: primary level (Grades 4–6) and lower secondary level (Grades 7–9). The quantitative data were complemented by focus group interviews with students in Grades 6 and 7 to capture their subjective perspectives and provide a more detailed picture. The findings from this project aimed to contribute to the development of prevention and intervention strategies to mitigate students’ alienation from school.

This paper focuses on qualitative data collected from focus groups conducted in the Canton of Bern, Switzerland, between 2015 and 2018. The focus groups aimed to capture students’ subjective perspectives and experiences related to school and learning, with particular attention to the transition to lower secondary school. Within the broader mixed-methods design of the SASAL project, the qualitative component fulfilled a distinct and complementary function: it enabled us to examine how students themselves articulate concrete school situations as alienating or (un)just. Such contextualized, experience-proximal accounts cannot be captured solely through questionnaires and therefore provide a crucial interpretive foundation for subsequent quantitative analyses conducted with the same cohort.

The sample included students from both primary and lower secondary levels across two different school districts in Switzerland, each comprising one primary school and one lower secondary school; teachers and guardians voluntarily provided consent for students’ participation in the focus groups. Data collection occurred in Grade 6 (the final year of primary school: average age 12 years) and after the transition to Grade 7 (the first year of lower secondary school: average age 13 years). A total of 15 focus groups (*N* = 134) were conducted: eight at the primary level (*n* = 69, 42 female students and 27 male) and seven at the lower secondary level (*n* = 65, 37 female students and 28 male).

The focus group composition was determined by class affiliation, which fostered a degree of social familiarity among participants (Przyborski & Wohlrab-Sahr, 2021; Vogl, 2022). The transition to the highly stratified lower secondary level in Switzerland involves assignment to specific school tracks based on ability, resulting in new class compositions (Hascher & Morinaj, 2023). Because students were first interviewed in Grade 6 (i.e., prior to track allocation) and again in Grade 7 (i.e., after allocation), all performance levels of the lower secondary system were represented in the sample. Whenever possible, the same students were interviewed at both measurement points to maintain continuity, and approximately 57% of the participating students were interviewed twice.

### Data collection

Within the qualitative substudy of the SASAL project, focus groups were used as a communicative method to analyze the subjective perspectives of children and adolescents on alienating aspects of their school experiences. The conversational setting of the focus groups encouraged the exchange of individual and collective opinions and thus facilitated the identification of shared attitudes and experiences. This interactive process, characterized by the exchange of arguments, narratives, memories, and additional comments, provided a realistic means of capturing participants’ views (Przyborski & Wohlrab-Sahr, 2021; Vogl, 2022).

The discussions followed a semi-structured interview guide (Appendix 1) derived from the conceptual school alienation framework of Hascher and Hadjar (2018, 2021). This framework defined school alienation across three domains—learning, teachers, and classmates—and structured the thematic scope of the focus groups. Students were encouraged to reflect on hypothetical or experienced situations associated with school alienation. Students were intentionally not explicitly asked to discuss teacher justice. Rather, we wanted to understand whether school alienation is spontaneously connected to teacher (in)justice from students’ perspectives and experiences, and whether students’ discussions about school alienation would include specific forms of teacher (in)justice. To foster natural communication, the focus groups were conducted in Swiss German and followed guidelines to ensure comparability across groups, addressing key aspects of school alienation and its multidimensional nature. Each focus group lasted approximately 1 h. The verbal data were transcribed, anonymized, and translated into standard German for analysis.

### Data analysis

Data collected from the focus groups were subjected to a structured content analysis following Kuckartz (2016), using MAXQDA 2022 software. This method employs a systematic, rule-based procedure to condense and summarize the data (Kuckartz, 2016). Categories were developed through both deductive and inductive approaches in a multistage process. Initially, categories were derived from the research questions and the relevant literature, focusing on aspects of teacher justice that students cognitively perceived as negative and/or that were affectively linked to negative emotions. These categories were then iteratively refined to inductively develop subcategories. To ensure conceptual precision and to account for the distinction between fairness and justice outlined in the theoretical framework, the analysis followed a two-step coding procedure. In step 1, all student statements were categorized using the overarching category system (Appendix 2) to identify negatively perceived situations and experiences related to unfairness—that is, students’ global evaluative judgments of inappropriate or alienating classroom events (Cropanzano et al., 2015; Zhaleh et al., 2025). This allowed us to identify the experiential manifestations of perceived unfairness without presupposing specific justice dimensions. In step 2, the segments identified in step 1 were assigned to the corresponding justice codes, based on established criteria for distributive, procedural, and interactional justice (Appendix 3). Whereas step 1 indexed fairness at the descriptive level of students’ experiences, step 2 mapped these experiences onto theoretically defined justice principles. This sequential procedure ensured that fairness experiences served as the empirical anchor, while justice categories provided the normative structure needed to differentiate the underlying rules, criteria, and teacher behaviors that informed students’ perceptions.

Coding was conducted by two independent researchers trained in qualitative content analysis who were not involved in the students’ teaching or school contexts. Both coders were familiar with the theoretical framework of school alienation and teacher justice, but they did not participate in data collection, thereby reducing potential bias. To assess intercoder reliability, 20% of the dataset was coded independently, resulting in an acceptable Brennan and Prediger’s kappa coefficient of 0.82. After refining the category system, the full dataset was analyzed using this category system.

## Results

The analysis identified several alienating aspects that were related to students’ perceptions of teacher (in)justice. Students described sensitive aspects of teachers’ behavior that potentially contributed to the emergence and consequences of school alienation. These aspects were grouped into six domains:

1. Teacher–student relationship.
2. Peer conflicts.
3. Learning processes.
4. Assessments.
5. Disciplinary measures.
6. Allocation and transition.

### Perception of injustice in the teacher–student relationship

#### Highly hierarchical nature of the teacher–student relationship and teachers’ privileges (1)

Students described the teacher–student relationship as highly hierarchical and emphasized that teachers enjoyed privileges that were not available to students. They expressed a desire for more mutual communication and opportunities to provide feedback, for example, by evaluating teachers as part of a reciprocal process. As one student noted, “I think it would be cool if you could also evaluate the teachers, because they evaluate us the whole time and then maybe they could improve their teaching a bit.” (F44). Students perceived it as unjust when teachers granted themselves privileges such as arriving late for class and then justified these behaviors with excuses they considered illegitimate. Such behaviors were interpreted as violations of procedural and interactional justice, particularly through inconsistency, double standards, and unequal rule enforcement. Interpersonal mistreatment further reinforced students’ feelings of relational devaluation.

#### Violations of conventions of social behavior (2)

Students reported that teachers violated basic conventions of social behavior, for example, through disrespectful remarks, ironic comments, or shouting at students in front of the class. One student described such an incident: “[The teacher] just shouted at one person, really shouted at him in front of the whole class and also insulted him, at least I think so. For example, that the family doesn’t even know what school is and … things like that aren’t right, I’m sorry, but things like that aren’t right.” (F46). Students also perceived teachers as unreliable, lacking empathy, and as insufficiently attentive to students’ needs and everyday realities. Additionally, they felt that some teachers prioritized their own interests and positioned themselves at the center of interactions. As role models, teachers were expected to display emotional stability; however, moodiness or impulsive behavior was described as unsettling. Particularly damaging were experiences of insults, humiliation, embarrassment, and supposedly humorous defamation. Overall, these accounts reflected violations of interactional justice, especially through disrespect, shaming, and emotional outbursts. Procedural justice concerns appeared only marginally and were overshadowed by broader violations of professional and ethical standards.

#### Preferential treatment of certain students (3)

Students reported experiences of preferential treatment when teachers visibly favored certain individuals or groups. They noted that teachers’ personal preferences influenced interactions and shaped how students were treated. This perception was expressed, for example, in the view that “a teacher must manage not to have favorite students” (M26), together with the acknowledgment that “yes, they can have favorite students — but [should] treat everyone the same” (F41, F39). Students highlighted procedural injustice, particularly in relation to unequal treatment, partiality, and inconsistently applied standards that were influenced by teachers’ personal preferences.

#### Summary of identified aspects and school-level differences

**Fig. 2:**
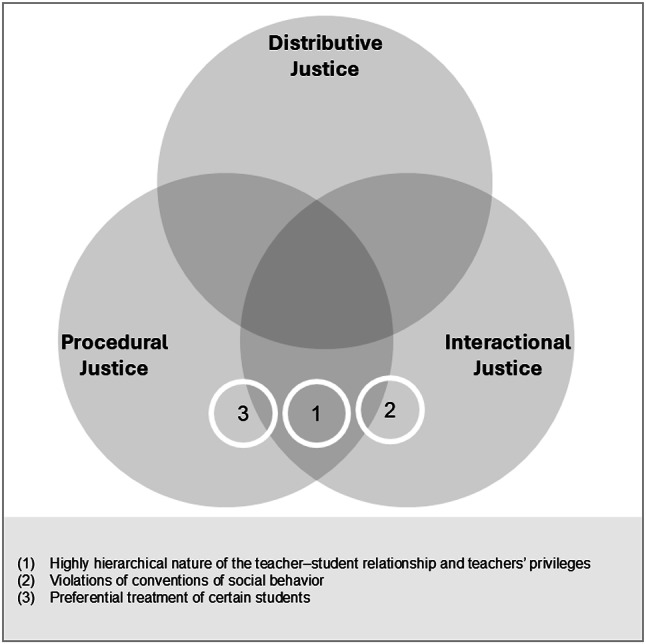
Justice dimensions in the teacher–student relationship

These alienating social aspects of interactions with teachers were associated with students’ perceptions of teacher justice within the teacher domain of school alienation. Figure 2 summarizes the identified aspects and their positioning within the three justice dimensions. The findings for the teacher–student relationship applied equally to students’ experiences at both the primary and lower secondary levels.

### Perception of injustice in relation to peer conflicts

#### Standing apart and ignoring peer conflicts (1)

Students described it as problematic when teachers remained passive during peer conflicts, particularly when students sought assistance in resolving them or were visibly overwhelmed. One student summarized this inconsistency, stating that “she always interfered when you didn’t ask her and when you did ask her, she didn’t do anything.” (F23). Such accounts reflected perceived violations of procedural justice, especially regarding consistency and ethicality. Students perceived the teacher’s inconsistent responses and the lack of professionally adequate support as central aspects of injustice in the handling of peer conflicts.

#### Insufficient, inadequate, imprudent prevention, and intervention measures in peer conflicts (2)

Students in the focus groups reported that some teachers intervened in peer conflicts without sufficient reflection or situational awareness. They described inadequate or imprudent prevention and intervention measures, noting that even minor disputes were sometimes addressed extensively, which could unintentionally intensify the conflict. As one student expressed, “teachers make the situation much worse than it actually is … They then exaggerate everything instead of just saying: Now you sort it out yourselves” (F6). Students further criticized delayed interventions that disrupted ongoing resolution processes or reignited conflicts that had already begun to fade. Intervening without being asked was perceived as patronizing, as it deprived students of opportunities to resolve issues independently. They also reported misinterpretations of conflicts when not all perspectives were considered or when relevant background information was ignored. In certain instances, preventive measures—such as excessive sensitization—were regarded as exacerbating rather than alleviating conflicts. Overall, these dynamics reflected violations of procedural justice, particularly regarding accuracy, consistency, and bias suppression. Students felt that interventions were often based on incomplete information and were pedagogically inappropriate or ineffective, contributing to their perception of teachers’ actions as unjust.

#### Favoritism and partiality (3)

Students also reported that teachers acted with favoritism and partiality when managing peer conflicts. They perceived discrimination when certain students or groups—such as girls—received preferential treatment during conflicts, or when teachers took sides and shielded alleged victims. One student noted, “sometimes it seems to me a bit, it’s a bit like the teacher takes a side” (F11). These accounts reflected concerns related to procedural justice, with bias suppression as the central issue. Students described situations in which teachers relied on incomplete information, involved students unevenly in clarification processes, or responded with pedagogically questionable actions. Interactional and distributive aspects appeared only marginally and they generally arose indirectly from perceived partiality.

#### Conflict resolution and clarification processes during lessons and involvement of additional professionals (4)

Students perceived conflict resolution and clarification processes conducted during lessons as unjust, particularly because they resulted in a loss of valuable learning time for uninvolved classmates. As one student explained, “you want to learn when it’s class time and not have to clarify things for the others when you have nothing to do with it” (F49). They also criticized the involvement of additional professionals, such as school social workers, which students often experienced as inadequate or excessively time-consuming. Overall, these perceptions were primarily linked to distributive justice, especially regarding the need dimension, as the loss of instructional time and the inclusion of uninvolved peers were viewed as inequitable. Procedural justice concerns also emerged, particularly regarding representativeness and ethicality, when students felt their perspectives were insufficiently considered or when interventions appeared to them disproportionate or inefficient.

#### Summary of identified aspects and school-level differences

**Fig. 3:**
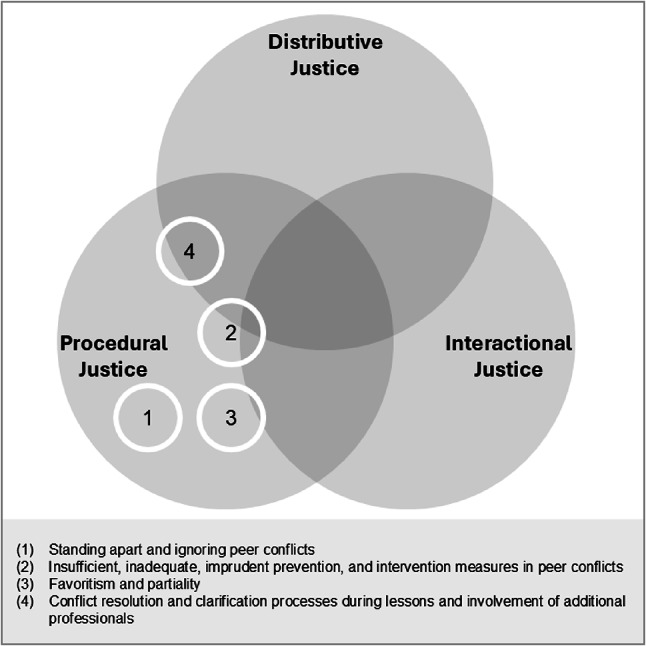
Justice dimensions in peer conflicts

Conflict situations among students had considerable potential to elicit perceptions of injustice in teachers’ actions. A comparison between primary and lower secondary levels revealed that lower secondary teachers tended not to devote sufficient time to addressing conflicts outside the classroom, largely due to the subject-teacher system, which entailed room changes after class. Figure 3 summarizes the identified alienating aspects and relates them to the three justice dimensions.

### Perception of injustice in relation to learning processes

#### Lack of professional competences to effectively promote learning processes (1)

The students reported that teachers failed to adequately respond to individual learning, developmental, or performance levels, which led to perceptions of injustice. One student illustrated this experience by saying, “And if you ask a question, sometimes she doesn’t know anything and … goes on to the next one without answering it. And if you then say afterwards, ‘ehm, I have another question,’ she simply says ‘now you’re quiet’, afterwards it goes on your record” (M26). Students felt inadequately supported in their learning processes, for example, when they were required to correct their work (e.g., worksheets) while generally receiving minimal formative feedback and lacking clear guidance on how to improve their work. They also criticized classroom climates that were not conducive to learning, often linked to weak classroom management. A lack of professional competence to effectively promote learning processes was also evident in the limited consideration of students’ needs. Students explained that individual interests and needs were sometimes disregarded, that they had no influence on the content and organization of lessons, and that teachers showed little flexibility when making decisions—such as determining group compositions in cooperative learning settings or acquiring materials to support learning—without involving them. Students also experienced pseudo-participation, meaning that jointly agreed decisions were delayed or not implemented. They also noted that democratic decision-making processes occasionally disadvantaged minority groups. Overall, these accounts primarily reflected distributive justice concerns, especially the unequal consideration of students’ needs. Procedural justice was also implicated when students experienced restricted opportunities for voice, inconsistent decision-making, or decisions made without an adequate informational basis.

#### Preferential treatment and discrimination in learning processes (2)

Students reported perceptions of injustice when teachers favored certain students or groups during learning processes. Despite comparable levels of competence, some students were called on more frequently, evaluated more positively, or given more opportunities to participate in decision-making within the classroom. Students described advantages for higher-achieving students, who often received more support, attention, and praise, while lower-achieving students were demotivated and disadvantaged by internal differentiation measures that offered only limited learning opportunities. These patterns were particularly evident at the primary level, which is characterized by high performance heterogeneity. One student described this distinction very explicitly, recalling that “the teacher also said that she was the teacher for the good ones and the rest should go to the teacher for integrative support later if they have any questions” (F23). At the primary level, perceived disadvantages could also be gender-specific; teachers were described as paying more attention to girls’ needs and calling on them more frequently. Students also observed inequalities between classes, with some receiving more extracurricular opportunities or greater freedoms than others. Overall, these experiences primarily reflected procedural justice concerns, particularly inconsistent application of rules and insufficient suppression of bias. Distributive justice was also implicated when unequal expectations, resources, or support appeared to disadvantage specific groups.

#### Inadequate explanations, support, and efforts to ensure understanding (3)

Students reported that explanations in class were insufficiently clear or not adapted to their individual learning levels, which they perceived as unjust, particularly at the primary level. One student described this experience, noting, “But if it’s something more complicated and you don’t understand it … then it’s almost your fault if you go and ask again—sometimes the answer is almost like ‘yes, you should have paid attention’” (M30). Students also criticized teachers for failing to check whether their explanations were understood or to consider alternative solution approaches. Students mentioned that some teachers lacked subject-specific expertise or were unable to point out certain topics, which resulted in long waiting times and an unnecessary loss of learning time and further exacerbated the problem when teachers delegated explanations to other teachers. Students also noted that explanations delivered exclusively in whole-class settings either overwhelmed lower-achieving students or slowed down higher-achieving ones. At the lower secondary level, students additionally noted that teachers had limited time during breaks due to the subject-teacher system and frequent room changes, making it more difficult to ask questions or receive support. Overall, distributive justice issues emerged when support, differentiation, or explanations were perceived as inadequate, while procedural justice concerns arose when certain peers appeared to receive more help or attention or when instructional decisions lacked a clear informational basis or were applied inconsistently. Students also reported interactional justice violations, such as disrespectful treatment or inappropriate remarks by teachers.

#### Lack of coordination and consideration of the student perspective on homework (4)

Students expressed perceptions of injustice regarding a lack of coordination and the limited consideration of their perspectives on homework. They reported having little influence over the amount or timing of homework and criticized short-notice or poorly coordinated assignments, which they associated with elevated stress levels and constraints on leisure activities. Students particularly emphasized insufficient coordination among subject teachers, especially when several assignments accumulated at once. They further noted that they preferred to complete learning activities during lesson time. Students also viewed homework that resulted from insufficiently structured lessons as unjust, especially when tasks that should have been completed in class were shifted to homework. One student summarized this sentiment by saying, “In German it’s a bit like this … we hardly do anything in class, but we have quite a lot of homework” (F37). Additionally, requiring lower-achieving students to complete unfinished classwork at home was perceived as overburdening and inequitable.

Overall, students’ perceptions primarily reflected procedural justice concerns, especially inconsistent practices and insufficient coordination among teachers. Distributive justice concerns emerged when students felt that their needs, workloads, or personal circumstances were not adequately considered or when homework demands lacked clear pedagogical justification.

#### Summary of identified aspects and school-level differences

**Fig. 4:**
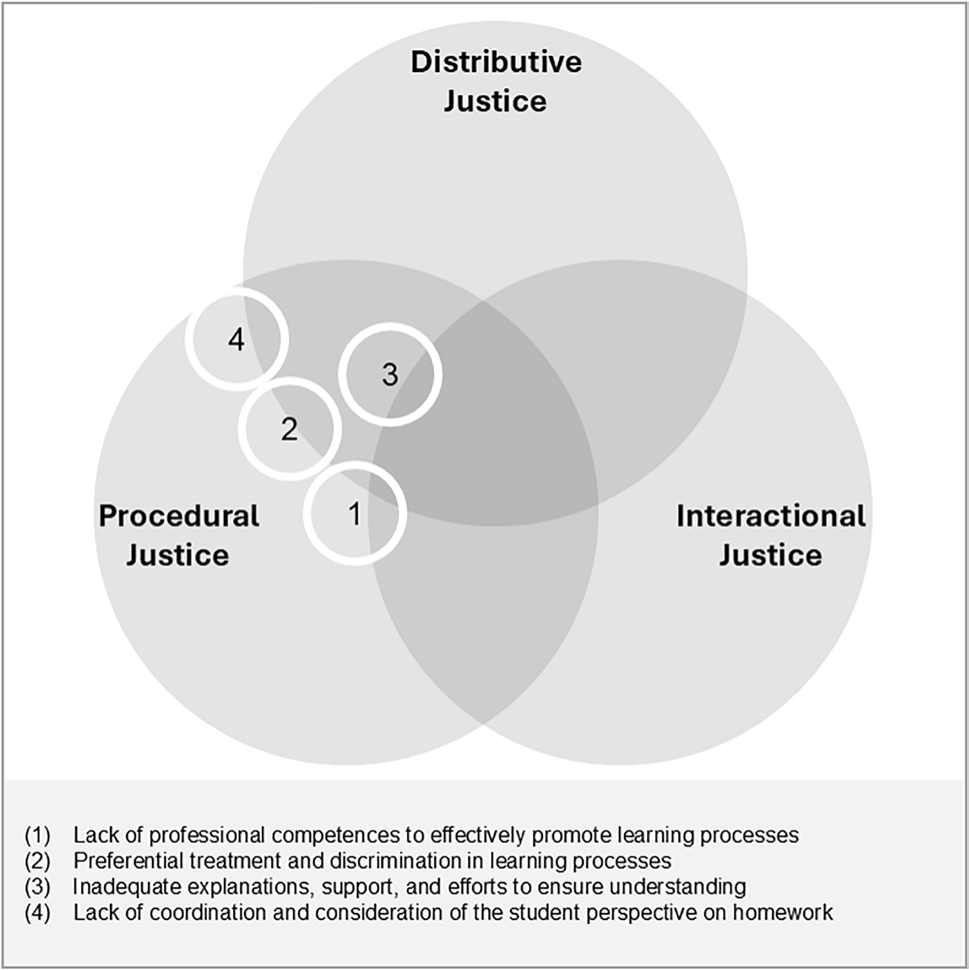
Justice dimensions in learning processes

Figure 4 summarizes the identified alienation-relevant aspects and assigns them to the corresponding justice dimensions. At the primary level, teachers faced pronounced performance heterogeneity, which contributed to perceptions of injustice regarding explanatory processes, the preferential treatment of students with higher achievement, and disadvantages for students with lower achievement. At the lower secondary level, the subject-teacher system complicated the coordination of instruction and student workload, resulting in reduced time for student support.

### Perception of injustice in relation to assessments

#### Lack of transparency and misleading information prior to assessment (1)

In our data, students exclusively referred to summative assessments when discussing assessment. Students reported that a lack of transparency and misleading information prior to assessments hindered adequate preparation. They described unannounced tests, assessments covering content only briefly addressed in class, and learning objectives that were formulated either too broadly or in excessive detail. Extensive assessments covering long learning units were also experienced as overwhelming. Students additionally criticized practice tests used for preparation that differed considerably from the actual assessment in content, structure, or level of difficulty. One student expressed this frustration by stating, “When she gives us the practice test, really, and afterwards she says that this is exactly what comes up and then you practice this, a few more examples and then afterwards in the test perhaps something else comes up” (F29). Encounters with unfamiliar task formats or complex authentic materials—such as non-simplified audio texts or unadapted source documents—further contributed to uncertainty. Overall, these experiences reflected procedural justice concerns, particularly perceived inaccuracies in test content and inconsistent or unclear assessment practices. Students’ accounts also reflected violations of informational justice, particularly regarding transparency and justification, as unclear or misleading communication about assessment content, criteria, or expectations limited their ability to prepare adequately.

#### Unequal treatment of students during assessment situations (2)

Students also reported experiences of unequal treatment during assessment situations. At the primary level, higher-achieving students received additional teaching materials to address performance heterogeneity within the class, while lower-achieving students were given assessments with reduced performance requirements. Students further perceived it to be unjust when some peers received more guidance, hints, or supportive comments during assessments. One student described this sense of unequal treatment: “I think it’s a bit daft of him … because last time, for example, I did the whole task wrong and he didn’t tell me anything” (F14). Students also emphasized the importance of a calm, supportive, and distraction-free assessment environment and considered it the teacher’s responsibility to ensure such conditions. Distracting or stressful situations were perceived as preventing them from performing at their best. Overall, students interpreted these experiences primarily through the lens of distributive justice, as differences in preparation materials, test difficulty, or in-test support were perceived as inequitable. Interactional justice concerns also emerged when teachers did not adequately justify differential treatment during assessments.

#### Lack of transparency in grading (3)

Students reported a lack of transparency in grading. They perceived it as problematic when overall scores were not displayed or when performance was coded only using symbols (e.g., smileys) instead of grades. At the primary level, students noted that class comparisons were often impossible because assessments were already differentiated in preparation for future ability grouping. Incorrect grading was also described as unjust, particularly when students noticed discrepancies between their grades and those of their classmates. In addition, considering work and learning behavior in grading was perceived as unjust because these aspects did not align with the learning objectives being assessed. At the lower secondary level, students reported a lack of learning progress despite increased effort, alongside unexplained declines in grades. One student described this experience as follows: “It’s just a bit strange when you used to score 5–6 [with grade 6 representing the highest achievement] in math in Grade 6 and then somehow you suddenly get a 3–4 for math. That was the case for me, you’re a bit shocked” (M13). Overall, these experiences reflected procedural justice concerns, particularly perceived inaccuracies, shifting standards, and insufficiently explained grading decisions. Students also described distributive justice issues when outcomes did not correspond to their efforts, as well as interactional justice concerns when teachers failed to provide clear explanations or justifications.

#### Performance-related embarrassment (4)

Students also described situations in which teachers publicly announced assessment results without prior consent. This practice led to feelings of performance-related embarrassment and was perceived as unjust, particularly for students who preferred to keep their results private. One student explained, “Then she simply read out the grades from everyone in the class. And I don’t really have a problem with that, but if there are others who would rather not, then she shouldn’t actually do that” (F5). Overall, these experiences primarily reflected interactional justice concerns, as students interpreted the public disclosure of assessment results as a violation of respect, privacy, and sensitivity. These perceptions overshadowed other justice dimensions and strongly shaped students’ emotional responses to assessment practices.

#### Insufficient follow-up on the assessment (5)

Students reported that they rarely received formative feedback after assessments, leaving errors unaddressed and hindering their learning progress. They also described situations in which teachers refused to return overall unsatisfactory assessments, which prevented students from reviewing their mistakes. Make-up or substitute assessments were likewise perceived as unjust when they differed substantially from the original version, particularly because there was no universal right to retake an assessment. One student illustrated this inconsistency: “Sometimes we can repeat the German test or some test … only because many are not good, but if I’m not good at math … I can’t repeat it then” (M24). Overall, students interpreted these experiences primarily through the lens of procedural justice, especially with concerns about perceived inaccuracies, inconsistent retake opportunities, and insufficient clarification of assessment results. The absence of accessible feedback and clear explanations also reflected informational justice concerns, as students lacked the justification and guidance necessary to understand their errors and improve future performance.

#### Summary of identified aspects and school-level differences

**Fig. 5:**
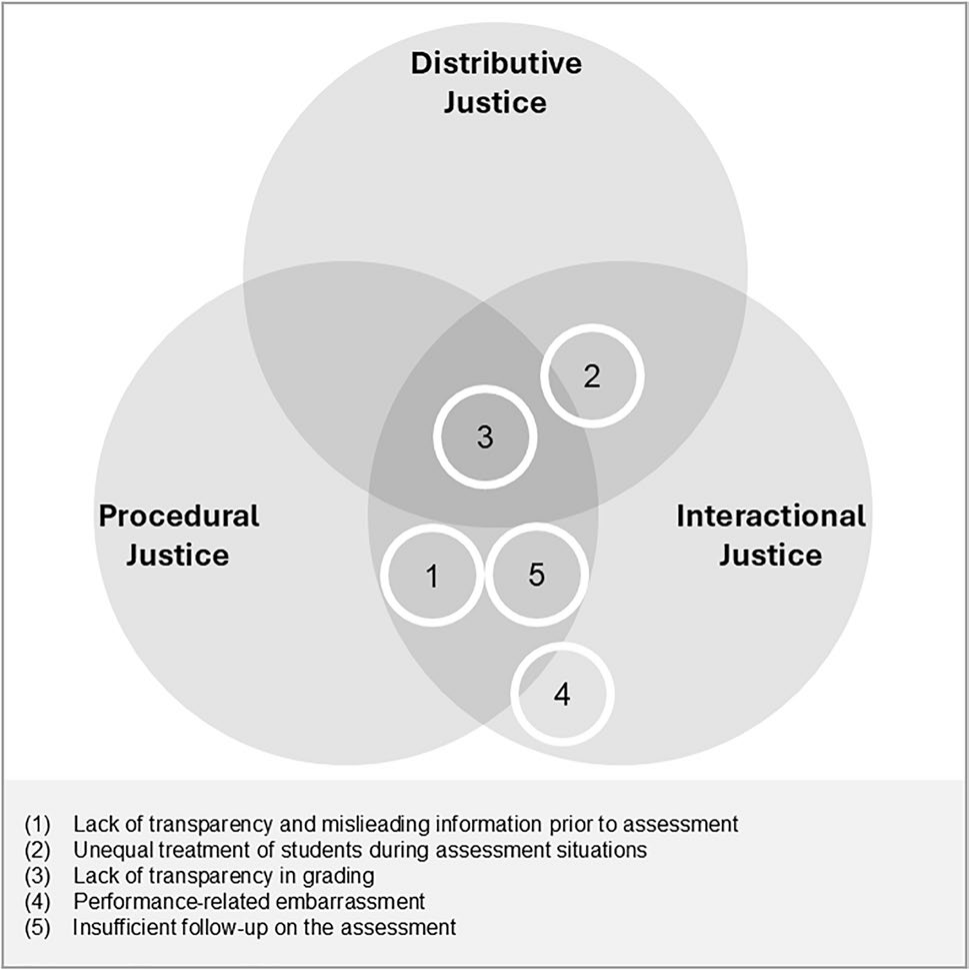
Justice dimensions in relation to assessments

Figure 5 summarizes the identified alienation-relevant aspects and assigns them to the corresponding justice dimensions. Several differences emerged between primary and lower secondary levels. At the primary level, dealing with pronounced performance heterogeneity contributed to perceptions of injustice, resulting in a lack of transparency and unequal conditions, such as disparities in learning materials and differences in teacher support during assessments. At the lower secondary level, students reported a perceived mismatch between their learning efforts and the grades they received, fostering a general sense that satisfactory grades were difficult to achieve.

### Perception of injustice in relation to disciplinary measures

#### Misinterpretations and inappropriate disciplinary measures (1)

Students reported that teachers sometimes misinterpreted situations and imposed inappropriate disciplinary measures. They felt that humorous or harmless, unintentional behavior was occasionally classified as punishable misconduct and that teachers failed to consider students’ individual needs or external circumstances, such as overload or excessive demands. Students also criticized teachers for not always reflecting on their role in escalating situations. As one student explained, “And then he tells his life stories that nobody is interested in, and if you don’t listen entirely, then you can just—go out the door, time-out” (M19). This issue was particularly salient in the accounts of students at the lower secondary level, where certain teaching methods or inconsistent classroom management could inadvertently provoke misbehavior. Students further described interventions as disproportionate, particularly when minor disruptions were sanctioned quickly or excessively. They noted that class teachers devoted more time to investigating the issue and engaging in dialogue with students, whereas subject teachers tended to impose disciplinary measures directly. Students perceived certain types of misbehavior, such as physical altercations, as leading to severe disciplinary measures without adequate reflection. Repeated minor transgressions, such as talking out of turn or arriving late, were sometimes met with increasingly harsh responses. Students also criticized instances in which teachers, especially those perceived as inexperienced, compensated for shortcomings in classroom management by imposing stricter disciplinary measures. Threats to inform parents or guardians were experienced as particularly unjust when used indiscriminately. As one student noted, “She is just always completely overwhelmed; afterwards there’s immediately a phone call home” (M30). Overall, students’ accounts reflected substantial procedural justice concerns, particularly regarding accuracy, ethicality, and consistency. They also described distributive justice issues when disciplinary actions did not consider students’ needs or the situational context, and interactional justice problems when teacher responses were experienced as disrespectful or emotionally charged.

#### Collective punishments, inadequate handling of sanction systems and school rules (2)

Students were critical of disciplinary measures that affected the entire class, particularly when the misbehavior of a few individuals resulted in consequences for everyone. For example, they perceived it as unjust when lesson time was extended or when teachers switched from group work to individual work as a form of collective punishment. As one student noted, “Yes, and the problem is that the whole class gets the punishment… because the others are behaving stupidly the whole time” (M39). Students also expressed concerns about the handling of sanction systems and school rules. They reported feeling pressured by sanction systems with automatic, fixed consequences and described psychosomatic symptoms associated with a reluctance to attend school. At the same time, students expected teachers to enforce sanction systems consistently, whereas inconsistent application was perceived as unjust. In bonus–malus systems, punitive elements were experienced as more salient because rewards were rarely emphasized. Although students generally accepted school rules as necessary regulations, they often perceived their implementation as inequitable when teachers interpreted, communicated, or enforced them differently. It was also perceived as unjust when teachers established classroom-specific rules that students believed should apply school-wide. Overall, students’ accounts strongly reflected procedural justice concerns, particularly regarding consistency, accuracy, and ethicality. Distributive justice issues emerged when collective measures failed to consider individual behavior or effort, while interactional justice concerns appeared more sporadically, mainly in relation to disrespectful communication.

#### Violation of dignity and unequal treatment regarding disciplinary measures (3)

Students described disciplinary situations in which they felt their dignity was violated or perceived unequal treatment. They considered it particularly unjust when teachers used personal insults or caused public embarrassment while enforcing disciplinary measures. Emotional or quick-tempered reactions from teachers were experienced as unsettling and anxiety-provoking, and they negatively affected the teacher–student relationship. One student described such an experience: “I was someone who talked quite a lot, and then he mimicked me… in a somewhat distorted voice and that made me a bit sad” (M29). Students also reported that teachers sometimes monitored certain individuals more closely or repeatedly corrected the same students, which they perceived as disproportionate and stigmatizing. They described cases in which isolated instances of misbehavior were overgeneralized and used to label a student more broadly. Unequal treatment was further perceived when similar types of misconduct received differing levels of sanctions or when students who were caught first were punished while others remained undetected. Students also mentioned that some vulnerable classmates were shielded by teachers and therefore received preferential treatment, whereas students who were more behaviorally salient or demonstrated lower academic achievement were sanctioned more severely. Students also perceived their class as being subject to stricter rule enforcement than other classes. Overall, these accounts most strongly reflected interactional justice concerns, particularly violations of respect through demeaning or publicly shaming interactions. Procedural justice concerns also emerged, especially regarding accuracy, ethicality, and bias suppression. In addition, the unequal distribution of disciplinary measures points to distributive justice concerns, particularly regarding equality and, in some cases, need-based considerations.

#### Summary of identified aspects and school-level differences

**Fig. 6:**
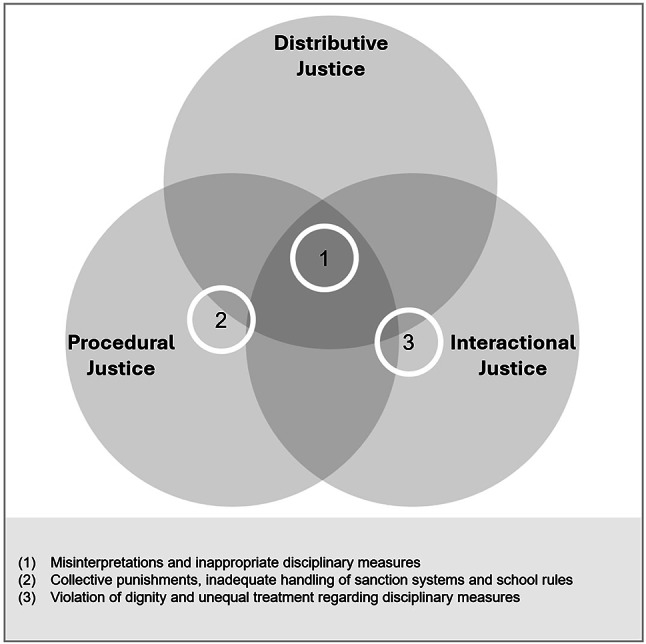
Justice dimensions in relation to disciplinary measures

Figure 6 summarizes the identified alienation-relevant aspects and assigns them to the corresponding dimensions of justice. At both school levels, disciplinary measures were the most frequently mentioned aspect of teacher justice associated with school alienation. No substantial differences emerged between primary and lower secondary levels, indicating that the identified issues were equally relevant across both levels.

### Perception of injustice in relation to allocation and transition

### Non-transparent, inadequate basis for decision-making, and lack of student involvement in the decision-making process regarding school transition (1)

Students identified various aspects of the teachers’ decision-making processes that they perceived as unjust. They described a non-transparent and inadequate basis for decision-making and a lack of student involvement in the decision-making process, emphasizing that they were only marginally involved and rarely consulted about their future placement. Unclear performance expectations made it difficult for them to assess their performance and anticipate their allocation. Students particularly criticized the focus on core subjects as the basis for decision-making, arguing that other subject-specific skills and interdisciplinary competencies were insufficiently considered. As one student explained, “Because these are three subjects and if you’re not doing so well in them, it means you’re not in the Sek [intermediate track]. So you could look at the whole thing a bit” (F15). When comparing their class with other classes, students also noted divergent requirements for teachers’ decision-making, such as discrepant performance requirements, workload, and the number of assessments. They found it unclear which time period or specific observation was considered or whether performance development was taken into account. If teachers modified the provisional allocation by ability during the process, students perceived this as arbitrary. They reported feeling insufficiently involved in the decision-making process, which was accompanied by feelings of powerlessness, especially when there was a lack of transparency about the timeline of the process. Some students noted that the decision was communicated in writing and the clarifying discussion occurred later, which they experienced as inadequate. Overall, these experiences primarily reflected procedural justice concerns, especially limited opportunities to express one’s views, unclear or inconsistent information, and concerns about accuracy. Distributive justice appeared less frequently but emerged in references to unequal conditions and perceived mismatches between evaluations and actual performance.

#### Performance pressure prior to allocation (ability grouping) (2)

At the primary level, students described strong performance pressure in the period leading up to the allocation to different ability tracks and felt that they received less support for their individual learning processes during this period. They noted that teachers used the allocation as a means of exerting pressure, which was accompanied by negative emotions, attempts to cover up perceived weaknesses, and reduced use of teacher support. Students reported that teachers emphasized the risk of being placed in a lower track, which heightened stress and contributed to this pressure. One student explained, “Our teacher told us all the time that yes, you can still fall down” (F18). Teachers further intensified pressure by implying that a lower-track placement could lead to lower educational, career, and life opportunities. Additionally, some teachers threatened to alter the provisional allocation in the event of performance stagnation or a decline in grades. Overall, these experiences primarily reflected interactional justice concerns. Students described teacher–student interactions as emotionally distressing or inappropriate and reported violations of respect when they felt belittled or publicly embarrassed. These emotionally charged interactions also intersected with procedural justice concerns, particularly around consistency, accuracy, and ethicality. Distributive justice issues also emerged when students perceived expectations or consequences as inequitable, particularly when the anticipated impact of allocation was viewed as disproportionately severe.

#### Summary of identified aspects and school-level differences

**Fig. 7:**
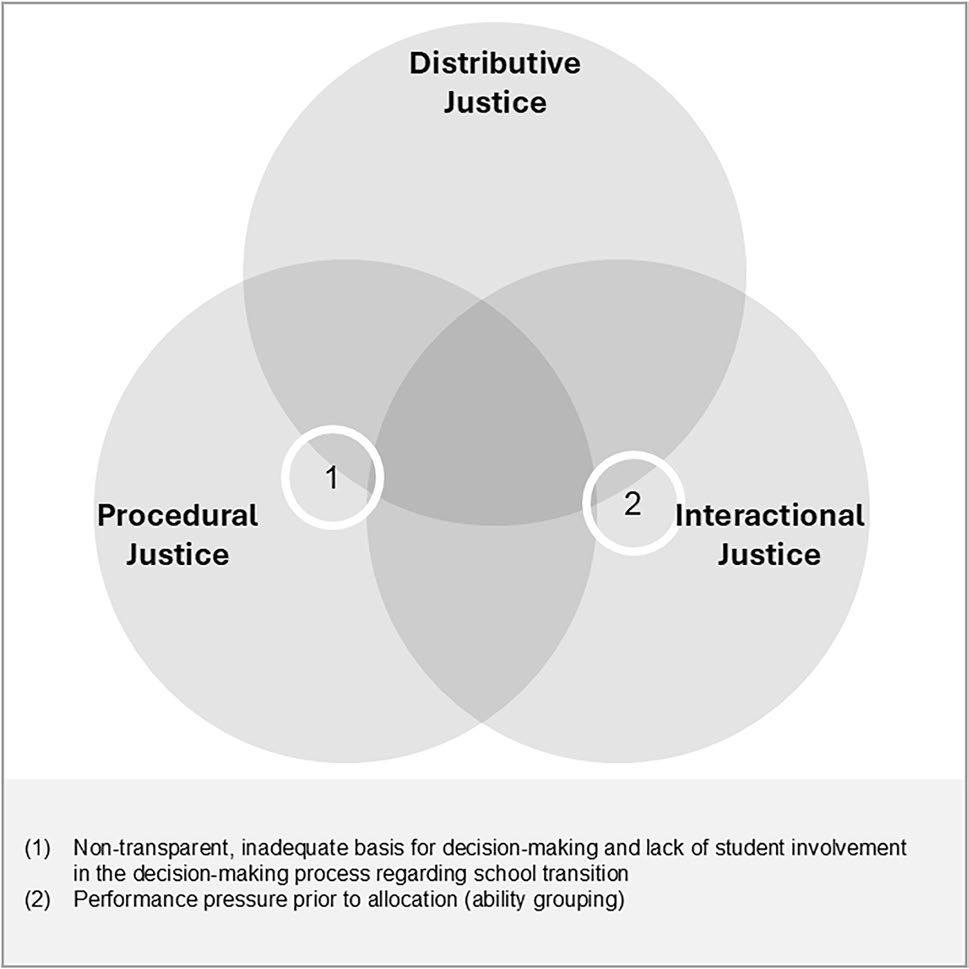
Justice dimensions in relation to allocation and transition

The allocation to different ability tracks as part of the transition to lower secondary education was associated with several alienating aspects that students perceived as unjust. Figure 7 summarizes these aspects and situates them within the corresponding justice dimensions. This issue emerged at the primary level and was linked to teachers’ decision-making processes, communication practices, and the use of allocation-related pressure.

## Discussion

This study examined how students describe the relation between school alienation and their perceptions of teacher justice across primary and lower secondary education. School alienation—a generalized negative attitude toward school involving both social and academic aspects—develops through recurring adverse school experiences, may intensify over the course of schooling, and is reflected in diverse consequences (Çağlar, 2013; Hascher & Hadjar, 2018, 2021; Hascher & Hagenauer, 2010). This article focused on perceived teacher justice, defined as students’ subjective perceptions of (in)justice in teachers’ actions and behavior (Dalbert, 2013; Dalbert & Stoeber, 2006), which may shape school alienation processes (Çağlar, 2013; Marcin et al., 2020).

Data were collected from two school districts in the Canton of Bern, Switzerland, and involved two measurement points: once during primary school and again during lower secondary school. In Switzerland, this transition represents a pivotal educational stage characterized by class changes, allocation by ability, and a shift from a class teacher system to a subject teacher system (Behrens, 2016; Hascher & Morinaj, 2023). These structural changes introduce various stressors that may heighten students’ negative perceptions of school. Although some students also articulated perceptions of structural or system-level injustice—particularly in relation to allocation procedures and the level-separating structures of lower secondary education—these aspects served primarily as contextual background. In line with the present definition of teacher justice, the analysis focused on perceptions directly linked to teachers’ actions and behavior. Against this backdrop, the study addressed three research questions: How are the three domains of school alienation associated with students’ perceptions of teacher (in)justice (RQ1)? Which dimensions of teacher (in)justice are related to the three dimensions of school alienation (RQ2)? And how do these associations differ between primary and lower secondary school students (RQ3)?

Given the study’s exploratory qualitative design, the findings cannot determine whether students who perceived greater injustice were the same students who reported stronger school alienation. Instead, the results illustrate how students interpreted and articulated possible intersections between the two constructs. The following discussion addresses the three research questions and situates the findings within existing theoretical and empirical work.

### Aspects of teacher justice related to the various domains of school alienation (RQ1)

School alienation and teacher justice share characteristics of a processual nature, are based on subjective perceptions, and are shaped by contextual influences (Hascher & Hadjar, 2018, 2021; Hascher & Hagenauer, 2010; Peter et al., 2013b; Resh & Sabbagh, 2014) and thus exhibit considerable complexity and dynamism. From the perspective of group value theory (Lind & Tyler, 1988; Tyler & Lind, 1990), both constructs relate to students’ fundamental need for belonging: respectful and dignified treatment signals inclusion, whereas perceived injustice conveys social devaluation and may foster alienation. School alienation consists of three domains—teachers, classmates, and learning—which manifest in different ways and thus produce multiple forms of school alienation (Hascher & Hadjar, 2018, 2021).

**Fig. 8:**
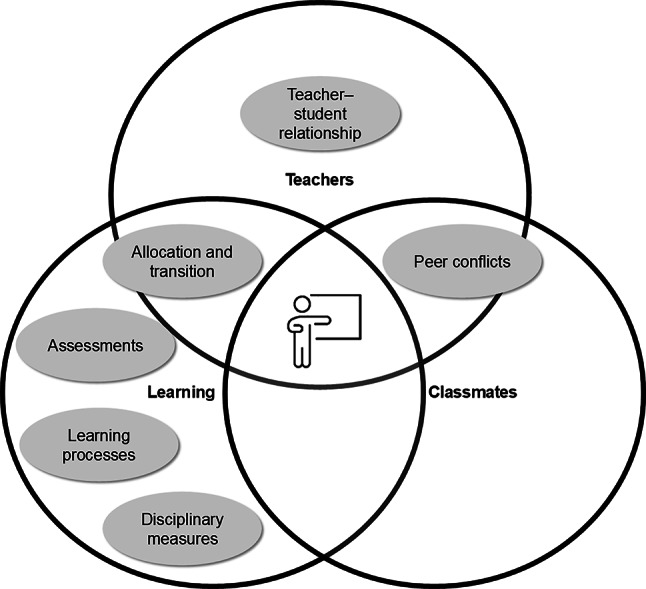
Areas relevant to school alienation and teacher justice (own illustration)

Addressing RQ1, our analyses identified student-reported experiences of injustice and mapped these onto the three domains of school alienation (see Fig. 8), illustrating how justice-related concerns may manifest within each domain.

In the *teacher domain*, students described experiences of interpersonal injustice, including insults, humiliation, and derogatory humor, which contradict normative expectations for respectful teacher conduct (Dalbert, 2013; Looker, 2022). The absence of reliability, empathy, genuine interest, and recognition of student needs further appeared to contribute to teacher-related alienation. Students also perceived preferential treatment and the hierarchical nature of teacher–student relations as unjust, which they felt may reinforce feelings of exclusion and hinder the development of positive relationships (Aegerter et al., 2023; Brown et al., 2003b; Buzzai et al., 2022; Looker, 2022; Schmid et al., 2021). This is consistent with the group value theory (Lind & Tyler, 1988; Tyler & Lind, 1990), which posits that respectful and equitable treatment signals inclusion and social value, whereas perceived injustice communicates marginalization within the group. Such signals of devaluation may therefore intensify experiences of alienation.

In the *classmates domain*, teachers play a critical role in shaping peer relationships, which gain importance during adolescence (Eccles et al., 1996; Morinaj et al., 2023). Students reported that insufficient, inadequate, or imprudent prevention and intervention in peer conflicts were perceived as unjust. Misinterpretations of conflict, favoritism, or the involvement of unrelated students and professionals were described as factors that may undermine both peer relationships and student–teacher relations, thereby potentially fueling alienation in this domain. Consistent with procedural justice, effective prevention and intervention should be just, impartial, accurate, professional, and equitable, while also encouraging student agency (Leventhal, 1980; Lotz et al., 2013; Peter & Dalbert 2013).

The *learning domain* emerged in students’ accounts as the most sensitive domain of school alienation. This underscores the importance of designing a school learning environment that enhances students’ well-being and supports effective learning and academic performance (Hascher & Hadjar, 2018, 2021; Hascher & Hagenauer, 2020). Teachers play a central role in ensuring justice in learning processes, assessments, disciplinary measures, and during allocation and transition. Students emphasized the importance of addressing individual learning needs, providing opportunities for co-determination and student agency, and ensuring structured and transparent teaching units in line with principles of constructive alignment. Didactic and subject-specific professional skills, as well as classroom management competencies, were described as crucial for establishing a positive classroom climate and thus a favorable learning environment. Clear objectives and formative feedback were viewed as essential for learning progress. Perceptions of injustice arose when favoritism or discrimination occurred—for example, unequal attention, praise, or support—and when external factors such as excessive workload or academic demands were insufficiently considered by teachers. In cases of misconduct, students pointed out the requirement for accurate and just disciplinary procedures, reflecting the procedural dimension of justice. Across all aspects, social relationships—especially the teacher–student relationship—remained of central importance.

### Dimensions of teacher (in)justice related to school alienation (RQ2)

Addressing RQ2, our findings suggest the multidimensional nature of teacher (in)justice and its perceived relevance for all domains of school alienation. Students’ perceptions indicated overlaps between the three justice dimensions—for instance, between distributive and procedural justice in relation to teachers’ limited time resources when addressing peer conflicts. Nevertheless, the data also point to typical and distinguishable characteristics of each justice dimension, which appear to align with theoretical assumptions drawn from organizational justice theory (Lind & Tyler, 1988).

*Distributive justice* concerns seemed to emerge when students perceived unequal allocations of instructional support, time, attention, or recognition. Violations of equity principles became salient when students’ efforts were perceived as not being reflected in their grades (Dalbert, 2004; Deutsch, 1975). Students also noted unmet needs-based criteria, particularly when teachers failed to consider emotional burdens or learning difficulties. Beyond classroom interactions, teachers’ decisions about ability grouping and track placement were described as creating unequal opportunities for future success.

*Procedural justice* violations were frequently mentioned and appeared to correspond closely to Leventhal’s (1980) criteria. Students described inconsistent rule enforcement, insufficient investigation of conflicts, and disproportionate disciplinary measures as unjust. They also reported unclear or unpredictable procedures in grading and classroom management, which made it difficult to anticipate how decisions would be made and applied. Combined with limited opportunities for voice, such procedural ambiguity further undermined students’ perceptions of justice. These findings resonate with research showing that clarity, participation, and correctability are central to just educational procedures (Dalbert, 2013; Pnevmatikos & Trikkaliotis, 2012).

*Interactional justice*—comprising interpersonal and informational justice—emerged in students’ narratives as a critical and emotionally charged aspect of students’ school experiences. Reports of disrespect, sarcasm, public embarrassment, and volatile teacher reactions indicated violations of interpersonal justice principles such as respect, dignity, and propriety (Dalbert, 2013; Lotz et al., 2013; Peter et al., 2013b). At the same time, students frequently described informational injustice, particularly when explanations, criteria, or rationales were missing or vague. Inadequate communication during assessment and disciplinary procedures heightened uncertainty and appeared to amplify feelings of school alienation, consistent with evidence that transparent communication is essential for trust and engagement (Chory-Assad & Paulsel, 2004; Kazemi, 2016).

Taken together, the results suggest that each justice dimension may contribute in distinct yet complementary ways to students’ perceptions of justice. Interactional and procedural concerns were particularly salient in students’ accounts and appeared to be closely linked to their emotional and cognitive distancing from school, pointing to the importance of justice-sensitive teacher behavior for mitigating school alienation.

### Differences between primary and lower secondary school (RQ3)

Addressing RQ3, our analysis suggests several distinct aspects and areas of focus that differentiate the two educational stages. Primary school students appeared particularly sensitive to factors influencing school alienation and teacher justice as they approached the transition to lower secondary school. The allocation and transition constitute a stressful, critical phase accompanied by various emotions, underscoring the necessity for teachers to provide careful and personalized support during this period (Grecu et al., 2019; Marcin et al., 2020; Stern, 2012; Symonds & Hargreaves, 2016). However, the students interviewed reported feeling exposed to high performance pressure prior to the allocation, often perceiving it as instigated by teachers and driven by the awareness of the different opportunities available in vocational training and the job market resulting from allocation to a particular track. Students perceived nontransparent, internally differentiated instructional strategies and varying types of support for students with lower and higher achievement as unjustly privileging certain individuals or groups, which they felt fostered feelings of preferential treatment and discrimination. Given that students attach great importance to equal treatment (Peter et al., 2013b), which may be challenged by the didactic principle of differentiation (Steinherr, 2017), deviations must be made transparent to the students (Dalbert, 2013). Another significant aspect concerned students’ reports of a non-transparent, inadequate basis for decision-making and lack of student involvement in the decision-making process, especially concerning the criteria and timelines of the allocation. This perception was further intensified by the minimal involvement of students and their parents or guardians.

Due to the subject-teacher system at the lower secondary level, the students in the sample reported that explanations, support, and efforts to ensure understanding were often inadequate, because subject teachers typically leave the classroom immediately after lessons and have limited time available for additional assistance. For the same reason, they also described teachers as imposing disciplinary measures without thorough clarification, leading to interventions perceived as inappropriate in form and intensity, and further contributing to a reduced sense of attachment to teachers (Symonds & Hargreaves, 2016). Furthermore, the students interviewed indicated that when addressing misconduct, teachers did not appear to consider their own contribution and external factors—particularly professional shortcomings such as teaching methods and classroom management—that may contribute to student behavior. This perceived neglect was linked in students’ accounts to negative emotions. Another factor that seemed to exacerbate school alienation related to teacher justice was that students at the lower secondary level perceived a lack of transparency in grading and a mismatch between their efforts and the grades they received. This mismatch in effort–outcome ratios raised concerns about distributive justice (Chory-Assad & Paulsel, 2004), which were associated with heightened performance expectations at the lower secondary level.

### Limitations and future directions

The present study is subject to various limitations. The results presented were obtained from two school districts and do not represent a randomized sample. Nonetheless, they illustrate the importance of school alienation and teacher justice, as well as their interactions and overlaps, while highlighting sensitive areas within the school context. The strong relevance of and relation to the construct of teacher justice revealed through data analysis suggest that future research could benefit from incorporating more specific questions to explore students’ perceptions of justice in greater detail.

Furthermore, both constructs depend on individual, subjective perceptions as well as contextual factors (Hascher & Hadjar, 2018, 2021; Hascher & Hagenauer, 2010; Peter et al., 2013b; Resh & Sabbagh, 2014). The data originate from the Swiss canton of Bern, with level-separating school models. In this respect, the results cannot be generalized, yet they provide valuable information for further research projects. A broader investigation of other school contexts with different structures and cultural backgrounds could enhance understanding of school alienation and teacher justice.

Another limitation is that in the present study, focus groups were conducted with students from different regular school classes, not solely those displaying high levels of school alienation. Therefore, the results reflect the perceptions of students in general education settings; nonetheless, they offer valuable insights. Future research could benefit from focusing specifically on students exhibiting high levels of school alienation.

This study also underscores the need to further clarify the statistical relationship between school alienation and teacher justice, particularly regarding the strength and direction of their associations. Additionally, adopting a longitudinal perspective would be well suited to the dynamic, processual nature of these constructs and thus allow a systematic examination of their development over time.

### Implications for practice

Prior research in various countries has shown that students’ perceptions of school alienation and teacher justice are closely related to student well-being, school climate, individual behavior, learning, and academic performance (Çağlar, 2013; Grecu et al., 2022; Hascher & Hadjar, 2018, 2021; Hascher & Hagenauer, 2020). Against this background, the findings of the present study suggest several implications for educational practice. These findings indicate the need to raise awareness among teachers, school psychologists, and other educational professionals of the importance of recognizing and responding to justice-related experiences that may contribute to school alienation. Expanding knowledge about school alienation and teacher justice can enable responsible professionals to carefully assess and adapt prevention and intervention measures to specific contexts and circumstances. To achieve this, effective multiprofessional collaboration is essential and can support the development of coherent, transparent, and responsive school structures. The topic holds significant relevance for (multiprofessional) educational teams. A deeper understanding of school alienation, particularly in relation to perceptions of injustice, is essential for shaping the school environment as a positive and meaningful experience for students. Leveraging synergies and diverse professional expertise can aid in identifying and addressing negative individual school experiences related to school alienation and teacher justice. This includes strengthening justice-sensitive teaching practices—such as clear communication, consistent procedures, and balanced support—to prevent the escalation of justice-related concerns. Such practices enable timely intervention with appropriate measures to counter the school-alienation process. At the same time, teachers may face tensions between different justice principles (e.g., equality and need), which cannot always be fully reconciled in everyday classroom practice. Moreover, even well-intentioned actions guided by justice principles do not automatically ensure that students will perceive them as just. Acknowledging these complexities, schools can still strive to become a safe, just, and joyful place of learning, characterized by positive, stable relationships and therefore a valuable individual resource (Donat et al., 2014, 2018; Peter & Dalbert 2013).

Particular attention must be paid to building positive and trusting relationships between teachers and students, especially at the transition to the lower secondary level, which is characterized by an increasingly negative perception of teachers (Eccles et al., 1996; Symonds & Hargreaves, 2016). Supporting students and families with transparent communication during allocation, differentiation, and assessment processes can help prevent experiences of injustice at this critical juncture. Teachers should reflect on their behavior towards students and engage in open exchanges with them to promote and make social interactions more explicit (Dalbert, 2013; Looker, 2022). This also includes careful guidance and support from teachers on peer interactions, especially because peer relationships become increasingly important as children develop and represent a valuable social resource (Morinaj et al., 2023).

### Conclusion

In sum, this qualitative study offered nuanced insights into how students described experiences of (in)justice across multiple domains of school alienation in primary and lower secondary education. By linking students’ accounts to established justice dimensions, it indicated that interactional and procedural aspects of teacher behavior are especially central to students’ emotional and cognitive distancing from school, while distributive concerns are particularly salient around assessment, differentiation, and track allocation. The findings underscore the importance of justice-sensitive classroom practices and relational work, particularly during educational transitions. They also highlight the value of systematically integrating student perspectives when designing school structures and support systems. Future research can build on these insights by examining the longitudinal interplay between teacher justice and school alienation and by testing targeted interventions aimed at strengthening just, supportive, and participatory school climates. Promoting justice-sensitive practices requires navigating competing principles and acknowledging that perceptions of fairness are inherently subjective. Thus, fostering justice in schools is not a matter of applying fixed rules but of continuously balancing contextual demands, professional judgment, and students’ diverse perspectives.

**Table 1:** Chapter overview of the focus group guidelines

Primary school	Lower secondary school
1 Teachers and schooling	1 Transition to lower secondary school
2 Class and classmates	2 Learning and schooling
3 Prevention from school alienation	3 Teachers
4 Transition to lower secondary school	4 Class and classmates
5 Motivation and goals	5 Prevention from school alienation
	6 Motivation and goals

**Table 2:** Overview of guidelines and content aspects for primary level

Primary school		
Chapter	Example: guiding question/narrative prompt	Aspects covered in this chapter
Introduction, icebreaker	Students select individually an appropriate emoji sticker (laughing, happy, sad, grumpy/annoyed, neutral) for each poster.	Poster 1: My teachers Poster 2: My class Poster 3: Learning and schooling
T1 Teachers and schooling	Can you tell us about a situation in which you found the lessons bad and felt uncomfortable?	Support Competence support, autonomy support, etc.) Teacher-student relationship/s Description of teaching situations Characteristics of the teaching Participation and disengagement
T2 Class and classmates	Let’s do a brainstorming session: What words come into your mind when you think of your class?	Description of the class Class climate, sense of belonging Friendship Conflicts
T3 Prevention of school alienation	Imagine Andreas and Tina don’t want to go to school anymore and just think school is stupid. What do you think could be the reason for this?	Support/Who Support/What Validation question (concrete examples) Well-being
T4 Transition to lower secondary school	In a few months, you’ll be going to lower secondary school. How are you feeling about that?	Expectations Selection procedures Needs Decision, decision-making Support
T5 Motivation and goals	When do you think you can be happy with yourself at school?	Sense of purpose in relation to learning and school Motivation Concept of a good student Expectations Grades Coping strategies in relation with academic failures Goals
Conclusion, closing phase	Imagine a fairy grants each of you a wish - you can wish for whatever you want for the future. What do you wish for?	Further aspects Questions that still need to be clarified

**Table 3:** Overview of guidelines and content aspects for lower secondary level

Lower secondary school		
Chapter	Example: guiding question/narrative prompt	Aspects covered in this chapter
Introduction, icebreaker	The students individually locate their mood on a mood barometer on various topics.	Poster 1: My teachers Poster 2: My class Poster 3: Transition to lower secondary school Poster 4: Learning and schooling
T1 Transition to lower secondary school	You have been at lower secondary level for a few months now. How have you perceived the first few weeks at your new school? How do you feel about your new school?	Transition experiences Allocation/selection Post-transfer (hopes and fears beforehand) Development/perceived changes Support Recommendations for younger students Development of class spirit/class community
T2 Learning and schooling	Can you tell us about a situation in which you found the lessons bad and felt uncomfortable?	Description of teaching situations Characteristics of the teaching Participation and disengagement
T3 Teachers	You scored teachers (result of the mood barometer) for teachers. What is the reason for this?	Support (competence support, autonomy support, etc.) Teacher–student relationships Change of class teacher–subject teacher system
T4 Class and classmates	Let’s do a brainstorming session: What words come into your mind when you think of your class?	Description of the class Class climate, sense of belonging Friendship Conflicts
T5 Prevention of school alienation	Imagine Jan and Julia don’t want to go to school anymore and just think school is stupid. What do you think could be the reason for this?	Support/Who Support/What Validation question (concrete examples) Well-being
T7 Motivation and goals	When do you think you can be happy with yourself at school?	Sense of purpose in relation to learning and school Motivation Learning at school Concept of a good student Expectations Grades Coping strategies in relation with academic failures Goals
Conclusion, closing phase	Students write down a wish for their time at lower secondary level.	Further aspects Questions that still need to be clarified

**Table 4:** Overview of category system

School alienating aspects/situations related to teacher justice (cognitive, affective expressions/evaluations of the students)			
Teachers exert a *direct* influence on the perception		Teachers exert an *indirect* influence on the perception (representative function)	
Main categories	Sub-categories	Main categories	Sub-categories
Lessons/learning	High workload (e.g. lack of coordination between teachers) Co-determination in class (e.g. imbalance of interest groups in democratic decisions) Promotion/support (e.g. untransparent internal differentiation measures) Violating behavior (e.g. publicly naming individual weaknesses/learning deficits) Preferential treatment of certain students/groups (e.g. more praise or appreciation)	Lessons/learning	Support, assistance (e.g. time resources)
Allocation/transition	Allocation process (e.g. lack of involvement of students) Transparency of ability grouping (e.g. unclear allocation criteria) Promotion/support (e.g. selection in the focus, lack of individual support and promotion) Ability grouping as a means of exerting pressure (e.g. threat of downgrading to a lower level)	Allocation/Transition	Distribution of opportunities (e.g. limitation of career choice) Allocation school site/class (e.g. lack of transparency) School model (separation) (e.g. homogenization of the ability groups)
Disciplinary measures	Disciplinary measures and sanction systems (e.g. lack of a sense of proportion in measures) Collective disciplinary measures (e.g. rewards linked to collective behavior in class) Misunderstandings (e.g. wrong students are punished) Contributory negligence of the teacher (e.g. boring lessons) Discriminatory disciplinary measures (e.g. embarrassment in front of the class) Preferential treatment of certain students/groups (e.g. stricter punishment of boys)	Disciplinary measures	School rules (e.g. different interpretation and enforcement)
Assessments	Transparency of assessments (e.g. imprecision of learning objectives) (Internal) differentiation (e.g. assessment according to future allocation) Factors influencing grading (e.g. inclusion of working and learning behavior) Preferential treatment of certain students/groups (e.g. providing additional help during assessments for weaker students)		
Conflicts	Restraint/handing over responsibility (e.g. delegation to class council) Collective solution finding (e.g. integrating uninvolved parties into the process) Misunderstandings (e.g. disregard of different perspectives) Interference (e.g. overreaction and involvement of school social work) Preferential treatment of certain students/groups (e.g. strong positioning, partiality)		
Teachers	Quality of relationship (e.g. low interest in students) Role model (e.g. rule violations by the teacher) Moodiness (e.g. taking out bad moods on students) Violating behavior (e.g. mocking students) Preferential treatment of certain students/groups (e.g. clear sympathies/antipathies)		

**Table 5:** Overview of justice dimensions

Distributive justice	Equity	Outcomes are considered just when they reflect students’ individual performance, effort, or contribution. Justice is achieved when higher effort or achievement leads to more favorable outcomes.
	Equality	Fairness is defined as equal treatment or equal outcomes for all students, independent of differences in performance or need.
	Need	Justice is linked to providing differential treatment based on students’ individual needs, such as additional support, resources, or leniency.
Procedural Justice	Process Control (Voice)	Perceived justice derives from being able to express opinions, offer input, and participate in decision-making processes, even without influencing the final outcome.
	Decision Control	Justice depends on having actual influence over decisions or outcomes, such as choosing topics, formats, or participating in binding decisions.
	Consistency	Procedures, rules, and evaluation criteria must be applied uniformly across people and time. Inconsistencies are perceived as unjust.
	Bias Suppression	Justice requires impartiality and the absence of favoritism, stereotypes, or personal bias in evaluations and disciplinary actions.
	Accuracy	Decisions should be based on correct, complete, and up-to-date information. Perceived inaccuracies or careless judgments indicate unjustice.
	Correctability	Just systems allow students to challenge, question, or appeal decisions. Opportunities for review and error correction enhance perceived justice.
	Representativeness	Just procedures take into account the perspectives, interests, and circumstances of different student groups, ensuring inclusive decision-making.
	Ethicality	Procedures must align with moral and professional standards, safeguarding dignity and avoiding humiliation, harm, or ethically questionable practices.
Interactional Justice – Interpersonal Dimension	Respect	Just treatment involves polite, dignified, and considerate interaction. Disrespect, humiliation, or dismissive behavior signals interpersonal injustice.
	Propriety	Justice requires avoiding inappropriate, offensive, or degrading comments or behaviors (e.g., sexist, racist, or mocking remarks).
Interactional Justice – Informational Dimension	Truthfulness	Communication is fair when it is honest, sincere, and factually truthful. Concealment or misleading information is interpreted as unjust.
	Justification	Justice hinges on clear, transparent, and adequate explanations for decisions, rules, and evaluations. Lack of explanation or vague reasoning reduces perceived justice.

## Appendix 1: Overview of the guidelines

See Table 1.

## Appendix 2: Overview of category system

See Tables 2, 3.

## Appendix 3: Overview justice dimensions

See Tables 4, 5.

## Declarations

### Generative AI and AI-assisted technologies in the writing process

During the preparation of this work the authors used ChatGPT in order to improve readability and language. After using this tool/service, the authors reviewed and edited the content as needed and take full responsibility for the content of the publication.
